# The efficacy of vitamin D supplementation for irritable bowel syndrome: narrow scope and GRADE miss-interpretation

**DOI:** 10.1186/s12937-022-00833-6

**Published:** 2023-01-20

**Authors:** Mohamed Abuelazm, Basel Abdelazeem

**Affiliations:** 1 Faculty of Medicine, Tanta University, El Bahr St., Tanta, Gharbia Governorate 31111 Egypt; 2 McLaren Health Care, Flint, MI USA; 3 Michigan State University, East Lansing, MI USA

**Keywords:** IBS, Vitamin D, Irritable bowel syndrome, IBS-SSS, IBS-Qol

## Abstract

We read the article by Haung et al. that pooled the effects of vitamin D on irritable bowel syndrome symptoms and associated quality of life. However, the current review suffers from some methodological errors: inadequate search strategy; the grading of recommendations assessment, development, and evaluation (GRADE) miss-assessment; and miss-interpretation. Accordingly, addressing the emphasized limitations will lead to more robust findings and conclusions.

Dear Editor,

We read with great interest the recent review by Haung et al. [1] on the efficacy of vitamin D on the severity of symptoms and the quality of life in irritable bowel disease (IBS) patients currently published in your prestigious journal. Although we praised the authors’ efforts, we were concerned by some methodological issues.

First, the narrow and defective search strategy did not cover all the possibly includable studies that meet their inclusion criteria. The authors included four studies with 335 participants. While we searched the same databases with the same search terms until January 2022, as mentioned by the authors. However, our search yielded three more eligible studies [2–4] with 351 participants that should have been included in the current analysis. Including the missed eligible studies can significantly change the summary effect estimates of the outcomes of interest. To clarify, a recently published systematic review and meta-analysis that included the previously mentioned missed studies yielded different findings regarding the IBS severity scoring system (IBS-SSS) [5]. Another review that also included the missed article yielded consistent IBS-SSS findings [6]; however, its results have been under debate due to statistical concerns [7]. In addition, the narrow search strategy that leads to missing multiple key publications in the pooled analysis is a serious limitation of systematic reviews that have been previously emphasized [8, 9].

Second, the authors assessed the quality of evidence following the Grading of Recommendations Assessment, Development, and Evaluation (GRADE) [10]. However, we suspect a miss-assessment of the certainty of evidence. On one hand, the authors assessed IBS-SSS and IBS total score (IBS-TS) as “very low” quality of evidence downgrading it in three domains (inconsistency – imprecision – publication bias) assessed as “serious” in all of them. On the other hand, the authors assessed IBS quality of life (IBS-Qol) as “low” quality of evidence while downgrading it in four domains (risk of bias - inconsistency – imprecision – publication bias) assessed as “serious” in all of them, as demonstrated in (table 2) [1]. Surprisingly, IBS-Qol was downgraded more times than IBS-SSS and IBS-TS, but IBS-Qol’s certainty of the evidence was higher, which is contradictory with GRADE guidelines [10]. Also, the authors have not clarified the rationales for the downgrading of each domain.

Moreover, the authors failed to incorporate the certainty of evidence yielded by the GRADE approach to their conclusion. To clarify, the authors stated that vitamin D is effective for improving both the IBS-SSS and (IBS-Qol), but the certainty of the evidence of IBS-SSS was reported by the authors as “very low”, while the authors reported the certainty of the evidence of IBS-Qol as “low”. According to Chap. 15 of the Cochrane Handbook for Systematic Reviews of Interventions and the interpretation guidelines of the GRADE system [11, 12], “very low” and “low” rating implies that the evidence is very uncertain about the intervention effect and intervention may cause an effect, respectively. Hence, drawing a strong conclusion about the efficacy of vitamin D on IBS based on this weak evidence is questionable.

Altogether, the current review is undermined by the following methodological issues: narrow inadequate search strategy and GRADE system miss-assessment and miss-interpretation. The correction of the previously mentioned issues may lead to significant variations in the conclusions based on the current pooled analysis. Therefore, the findings and conclusions of the current review should be cautiously considered.

## Data Availability

Not applicable.
